# Relation between GSTP1 polymorphism and oxidative stress in patients with hepatocellular carcinoma

**DOI:** 10.1186/s43046-020-00049-x

**Published:** 2020-10-02

**Authors:** Shaimaa Gamal Hassan Elofey, Nevine F. Shafik, Noha Hassan Radwan, Osman Mohammed Mansour, Rasha Mahmoud Allam, Samia Shouman, Iman Attia AbdelGawad

**Affiliations:** 1 The Department of Clinical Pathology, Elkhanka Central Hospital, Qualiobeya, Egypt; 2 The Department of Clinical and Chemical Pathology, NCI, Cairo, Egypt; 3 The Department of Medical Oncology, NCI, Cairo, Egypt; 4 The Department of Biostatistics and Cancer Epidemiology, NCI, Cairo, Egypt; 5 The Department of Cancer Biology, NCI, Cairo, Egypt

**Keywords:** HCC, Glutathione, GSTP1

## Abstract

**Background:**

Glutathione can reduce the oxidative stress by converting the unstable to stable molecules and its status in hepatocellular carcinoma (HCC) is correlated with tumor growth and metastasis.

Glutathione S-transferase Pi (GSTP1) is reported to detoxify the xenobiotic substrates by catalyzing their conjugation to reduced glutathione (GSH) and its over-expression was demonstrated in the early stages of HCC, while loss of GSTP1 has been suggested to increase the risk of deoxyribonucleic acid (DNA) damage and mutation.

The aim of this study is to assess the relationship of GSTP1 polymorphism Ile105Val (rs1695 A > G) with HCC risk, and to investigate the oxidative stress status of HCC patients by measuring the antioxidant glutathione (GSH) levels.

This study was conducted on 99 newly diagnosed HCC patients and 80 apparently healthy individuals as a normal control group.

All participants were subjected to the measurement of plasma GSH levels according to Ellman’s method, and polymerase chain reaction-restriction fragment length polymorphism (PCR-RFLP) for the detection of GSTP1 polymorphismIle105Val (rs1695 A > G).

**Results:**

The occurrence of either the mutant homozygous or the mutant heterozygous genotype of GSTP1 was significantly higher in HCC patients, while the occurrence of the wild genotype was significantly higher among the normal control subjects.

Mutant GSTP1 genotype, older age, male gender, and high serum alanine aminotransferase (ALT) were associated with increased risk of development of HCC.

The best sensitivity, specificity, PPV (positive predictive value), NPV (negative predictive value), and overall diagnostic performance for plasma GSH at a cutoff level of 2003.5 μM/mg were 57.6%, 52.5%, 60%, and 40%. The area under the curve for GSH was 0.562.

**Conclusion:**

Mutant GSTP1 genotype was an independent prognostic factor for increased HCC risk which can be used in a risk assessment model for HCC.

Plasma GSH presents insufficient sensitivity and specificity for HCC.

## Background

Liver cancer was the sixth most commonly diagnosed cancer and the fourth leading cause of cancer death worldwide in 2018, with about 841,000 new cases and 782,000 deaths annually [1]. It is a major health problem with an increasing incidence in Egypt where it constitutes for 33.63% and 13.54% of all cancers in males and females respectively [2].

Both the immune response to viral hepatitis infection and oxidative stress are involved in the pathogenesis of HCC [3].

Storage of cysteine, anti-oxidant defense, and modulation of cell growth are some of the critical cellular functions induced by glutathione which is the main non-protein thiol in the mammalian cells. In HCC, GSH status is correlated with cellular proliferation, tumor growth, and metastasis [4].

There are 2 forms for glutathione: reduced (GSH) and oxidized (GSSG). The thiol group of cysteine in the reduced form is capable of donating a reducing equivalent (H^+^+ e^−^) to other unstable molecules, including reactive oxygen species. Donating an electron converts glutathione to the reactive state. This reactive glutathione can react with another reactive glutathione to form glutathione disulfide (GSSG), which can then be catalyzed by the enzyme glutathione reductase (GSR) to regenerate GSH [5]. That is how GSH can reduce the oxidative stress by converting the unstable to stable molecules.

Glutathione *S*-transferases (GSTs) family is composed of eight isoforms that are known to detoxify the xenobiotic substrates by catalyzing their conjugation to GSH. Glutathione S-transferase P1 (GSTP1) is the most studied isoform in cancer. GSTP1 over-expression was demonstrated in early stages of HCC [6], while loss of GSTP1 has been suggested to increase the risk of DNA damage and mutation [7].

Since GSTP1 was reported to protect cells from the effects of cytotoxic and carcinogenic agents, we suggest that GSTP1 polymorphism might be a risk factor for the development of HCC supported by some studies that reported the association between GSTP1 polymorphism and some types of cancer, including esophageal and colorectal cancer [8].

The aim of this study is to assess the relationship of GSTP1 polymorphism Ile105Val (rs1695 A > G) with HCC risk, and to study the relationship of the GSTP1 polymorphism with some clinico-pathological factors of HCC. Investigating the oxidative stress status of HCC patients by measuring the antioxidant glutathione (GSH) levels and assessing the relation between the glutathione level and the expression of GSTP1 were done.

## Methods

This study was approved from the ethical committee of the research institute Cairo University (Organization no. 0003381) under the number of 00004025 with a Federalwide Assurance (FWA) number 00007284 as well as the National Liver Institute-Shebein El-Kawm-El Monofeya. Written informed consent was obtained from all participants prior to inclusion in the study. This work was carried out in accordance with the Declaration of Helsinki for experiments involving humans.

This study was conducted on 99 newly diagnosed HCC patients who presented to the outpatients’ clinic of the surgical oncology department over a period of consecutive 22 months from November 2014 to August 2016. All patients were below the age of 57 years. They were proven to have HCC by computed tomography, magnetic resonance imaging with typical findings of HCC, or with biopsy. They were included in this study prior to any therapeutic intervention. Patients with any other concomitant malignancy or under medical or surgical treatment were excluded. The study also included 80 apparently healthy volunteers below the age of 57 years as a normal control group. They were 60 males and 20 females. Their age ranged from 34 to 56 years. Both groups were age and sex matched.

**Fig. 1:**
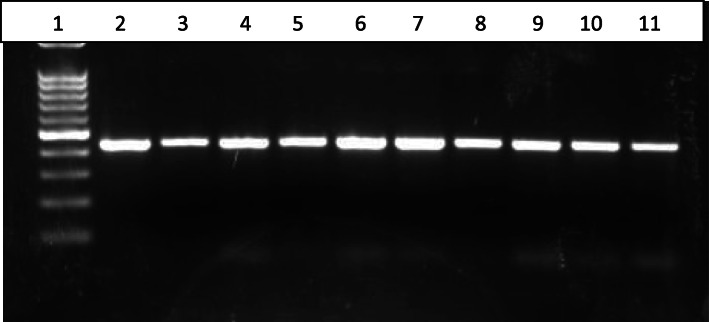
PCR product on agarose gel before digestion appearing as bands at 433 base pair on electrophoresis (lanes 2-11). Molecular weight marker (100 bp) (lane 1)

**Fig. 2:**
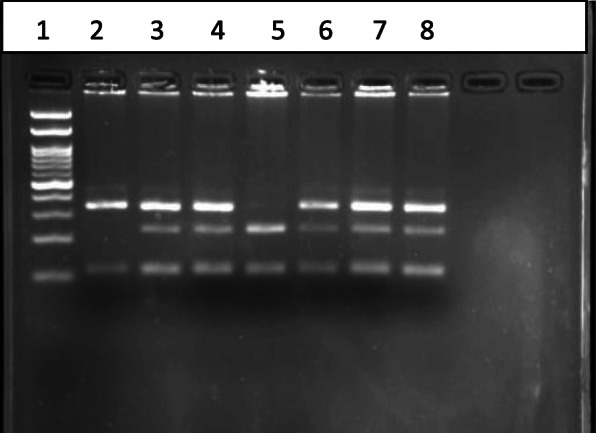
Digestion using ALW261 illustrated by agarose gel electrophoresis. Molecular weight marker (100 bp) (lane 1). Wild phenotype: If DNA is cut as 2 bands at 329 and 104 base pair (case 1) (lane 2). Mutant homozygous phenotype: If DNA is cut as 2 bands at 222 and 107or 104 base pair (case 4) (lane 5). Mutant heterozygous phenotype: If DNA is cut as 3 bands at 329, 222, and 107 or 104 base pair (case 2, 3, 5, 6, 7) (lanes 3, 4, 6, 7, 8)

All patients and control subjects were subjected to the following:

1. Careful history taking and clinical examination for patients.
2. Routine laboratory investigations: Complete blood count (CBC), liver functions tests, lactate dehydrogenase (LDH), urea, creatinine, prothrombin time (PT) and concentration (PC), and international normalized ratio (INR).
3. AFP (Alpha feto-protein) was done using ARCHITECT i1000SR chemiluminescent micro particle Immunoassay (CMIA) Analyzer, Abbott, USA. The ARCHITECT AFP assay is designed to have an imprecision of ≤ 7.5% within-laboratory (total) % CV for samples between 10 and 2000 ng/mL and an SD of ≤ 0.75 for samples less than 10 ng/mL down to the LoQ (i.e., 2.0 ng/mL).
4. Measurement of plasma GSH levels by spectrophotometry: Measurement of plasma GSH levels was done using spectrophotometry according to Ellman’s method. This method depends on the reduction of thiol reagent; Ellman’s reagent (5, 5′-dithiobis-(2-nitrobenzoic acid)) or (DNTB) by the sulfhydryl SH group in the GSH to form the yellow chromophore; 5-thiontrobenzoic acid, measured spectrophotometrically at 412 nm. Precipitation of protein thiols by trichloroacetic acid (TCA) was carried out before the addition of Ellman’s reagent. In a 10-ml glass centrifuge tube, 500 μl of heparinized blood was mixed well with 25 μl trichloroactic acid. The tubes were then centrifuged at 3000 rpm for 10 min at 4 °C. One hundred microliter of the resultant supernatant was mixed thoroughly with 850 μl of phosphate buffer, followed by addition of 50 μl Elleman’s reagent. After 5 min, the absorbance was measured spectrophotometrically at 412 nm against blank containing 100 μl distilled water. Plasma glutathione (GSH) content was calculated in comparison with GSH standard curve [9].

5. PCR-RFLP for the detection of GSTP1 polymorphism:

- Total genomic DNA was extracted from peripheral blood samples of patients with HCC and control subjects using QIAamp DNA mini isolation kit (QIAGEN) following standard procedures according to the manufacturer’s instructions.

- Genotyping of GSTP1 gene polymorphism Ile105Val (rs1695 A > G) was identified by polymerase chain reaction amplification and restriction fragment length polymorphism analysis (PCR-RFLP) [10]. The primers used were (5′-GTA GTTTGCCCA AGG TGAAG-3′) as a forward primer and (5′-AGCCACCTGAGGGGTAAG-3′) as a reverse primer.

- PCR was conducted with 100 ng of genomic DNA, 2.5 μl of 10× PCR buffer (Invitrogen, Carslbad, CA, USA), 0.25 μl of Taq DNA polymerase (Invitrogen, Carslbad, CA, USA), 1 μl of 10 mM/Ideoxyribonucleosides triphosphates (dNTPs) (Promega, Madison, WI, USA), and 1.5 μl of 10 pmol/ul of each primer. PCR was performed with 35 cycles of the following, 95 °C for 1 min, followed by denaturation at 95 °C for 45 s, annealing at 60 °C for 45 s, and extension at 72 °C for 1 min, with a final extension at 72 °C for 10 min. This resulted in one band at 433 bp (Fig. 1).

- Digestion of the PCR product with 10 U of Alw26 I (BsmAI) (New England Biolabs, USA) restriction enzyme in a final volume of 20 μl incubated at 37 °C overnight. The wild type genotype (I/I) produced a double band at 329 and 104 bp, whereas heterozygotes alleles (I/V) produced four bands at 329, 222, 107, and 104 bp. The homozygous polymorphic genotype (V/V) produced three bands at 222, 107, and 104 bp. Polymorphism was detected in 3% agarose gel (Fig. 2).

### Statistical methods

Data were analyzed using IBM SPSS advanced statistics, version 22 (SPSS Inc., Chicago, IL). Numerical data were described as median and range, while qualitative data were described as numbers and percentages. Chi-square (Fisher’s exact) test was used to examine the relation between qualitative variables as appropriate. Odds ratio was calculated using multiple logistic regression to assess the relationship of GSTP1 polymorphism with the HCC risk. Testing for normality was done using Kolmogorov-Smirnov test and Shapiro-Wilk test. Mann Whitney *U* test was used to compare median values of variables that were not normally distributed between 2 independent groups.

Comparing the median values of more than two independent groups was tested using Kruskal-Wallis test.

Receiver operating characteristics (ROC) curve was done to estimate the best cutoff point, then calculation of sensitivity, specificity, PPV, and NPV with their 95% confidence interval was done.

A *p* value ≤ 0.05 was considered significant. All tests were two tailed.

## Results

Patients’ characteristics are mentioned in Table 1.

**Table 1:** Descriptive analysis of HCC patients’ characteristics

Character		No. (%) (***N*** = 99)
**Age (years)**	**< 54**	55 (55.6)
	**55-56**	44 (44.4)
**Gender**	**Female**	24 (24.2)
	**Male**	75 (75.8)
**Family history of HCC**	**Negative**	87 (88)
	**Positive**	12 (12)
**Tumor size**	**≤ 3 cm**	36 (36.4)
	**> 3 cm**	63 (63.6)
**No. of focal lesions**	**≤ 3**	71 (71.7)
	**> 3**	28 (28.3)
**Stage (TNM classification)**	**I**	7 (7.1)
	**II**	19 (19.2)
	**IIIA**	30 (30.3)
	**IIIB**	15 (15.2)
	**IVA**	15 (15.2)
	**IVB**	13 (13.1)
**Stage**	**Early stage (I, II)**	26 (26.3)
	**Late stage (IIIA, IIIB, IVA, IVB)**	73 (73.7)
**Child score**	**A5**	21 (21.1)
	**A6**	32 (32.4)
	**B7**	18 (18.3)
	**B8**	10 (9.9)
	**B9**	15 (15.5)
	**C1**	3 (2.8)
**Distant metastasis**	**No**	87 (86.9)
	**Yes**	12 (13.1)
**Liver cirrhosis**	**Negative**	2 (2.1)
	**Positive**	97 (97.9)
**Portal vein**	**Thrombosed**	22 (25.0)
	**Patent**	77 (75.0)
**Splenomegaly**	**−ve**	38 (38.9)
	**+ve**	61 (61.1)
**Ascites**	Present	35 (35.3)
	Absent	64 (64.7)
**Lymph node metastasis**	**−ve**	76 (76.8)
	**+ve**	23 (23.2)
**HBV infection**	**B−ve**	96 (97.0)
	**B + ve**	3 (3.0)
**HCV infection**	**C−ve**	16 (16.2)
	**C + ve**	83 (83.8)
**GSH (μM/mg) (N: 684-2525)**	**Median**	1916.5
	**Range**	(862-3011)

The comparison between the HCC and normal control groups as regards GSTP1 phenotype shows that the frequency of GSTP1 GA and GG genotype were significantly higher in HCC patients compared to the normal control subjects (*p* = 0.001). The frequency of the homozygous GG genotype was also significantly higher in HCC patients compared to the normal control subjects (*p* = 0.003) (Table 2).

**Table 2:** Comparison between the HCC and the normal control groups as regards the glutathione S-transferase P1 (GSTP1) phenotype

			Group		***p*** value	OR	95% CI for OR
			Control (80)	Cases (99)			
**GSTP1**	**AA**	**N** (%)	60 (75)	45 (45.5)	< 0.001		
	**GA**	**N** (%)	20 (25)	44 (44.4)			
	**GG**	**N** (%)	0 (0.0)	10 (10.1)			
**Recessive model (GSTP1)**	**AA**	**N** (%)	60 (75)	45 (45.5)	< 0.001	3.6	Lower, 1.894
							Upper, 6.843
	**GA&GG**	**N** (%)	20 (25)	54 (54.4)			
**Dominant model (GSTP1)**	**AA&AG**	**N** (%)	80 (100)	89 (89.9)	0.003		
	**GG**	**N** (%)	0 (0)	10 (10.1)			
**GSTP1 (mutant versus wild)**	**Mutant**	**N (%)**	20 (25.0)	54 (54.5)	< 0.001*		
	**Wild**	**N (%)**	60 (75)	45 (45.5)			

*GSTP1* glutathione S-transferase P1

*Significant *p* value ≤ 0.05

Only the Child score (A), and absence of ascites were significantly associated with higher levels of plasma GSH (*p* value = 0.043 each) (Table 3).

**Table 3:** Comparison of the plasma levels of reduced glutathione with some prognostic factors of HCC

		GSH (μM/mg) (N: 684-2525)	Adjusted ***p*** value
		Median (range)	
**Age (years)**	**< 54**	1976 (862-3011)	0.885
	**≥ 55**	1861 (952-3000)	
**Gender**	**Female**	1861 (877-2625)	0.403
	**Male**	1952 (862-3011)	
**Tumor size (cm)**	**3**	2013 (990-2409)	0.891
	**Less than 3**	1952 (877-2720)	
	**More than 3**	1877 (862-3011)	
**No. of masses**	**≤ 3**	1921 (867-3000)	0.669
	**> 3**	1889 (862-3011)	
**Stage**	**Early stage**	1961 (867-3000)	0.778
	**Late stage**	1912 (862-3011)	
**Child score**	**A**	1991 (952-3000)	0.043*
	**B**	1952 (862-2720)	
	**C**	941 (894-987)	
**Distant metastasis**	**No**	1919 (867-3011)	0.333
	**Yes**	1247 (862-2931)	
**Liver cirrhosis**	**Negative**	2568 (2124-3011)	*
	**Positive**	1912 (862-3000)	
**Portal vein**	**Thrombosed**	1689 (894-2911)	0.859
	**Patent**	1988 (862-3011)	
**Splenomegaly**	**−ve**	1746 (910-3000)	0.455
	**+ve**	2003 (862-3011)	
**Ascites**	**Present**	1731 (862-2416)	0.043*
	**Absent**	1952 (922-3011)	
**L.Ns**	**−ve**	1914 (862-3011)	0.369
	**+ve**	2061 (899-2931)	
**Family history of HCC**	**Negative**	1912 (862-3011)	0.448
	**Positive**	2000 (992-2911)	
**HBV infection**	**B−ve**	1906 (862-3011)	0.237
	**B + ve**	2101 (2003-2573)	
**HCV infection**	**C−ve**	1838 (910-3011)	0.905
	**C + ve**	1921 (862-3000)	
**GSTP1 homo hetero vs wild**	**Mutant heterozygous**	1991 (877-2911)	0.198
	**Mutant homozygous**	1508 (894-2241)	
	**Wild**	1851 (862-3011)	
**GSTP1 wild vs mutant**	**Mutant**	1964 (877-2911)	0.569
	**Wild**	1851 (862-3011)	

Median and range in parenthesis

Significant *p* value ≤ 0.05

*GSH* reduced glutathione, *GSTP1* glutathione S-transferase P1

*No *p* value calculated because of small sample size

The comparison between the HCC and the normal control groups as regards different laboratory markers and plasma GSH reveals the presence of significant differences between both groups regarding ALT, aspartate transaminase (AST), alkaline phosphatase (ALP), albumin, urea, LDH, INR, hemoglobin (HB), total leucocyte count (TLC), and platelet count (*p* < 0.001) each, while no significant differences were found as regards GSH levels (*p* = 0.152) (Table 4).

**Table 4:** Comparison between the HCC and the normal control groups as regards different laboratory markers and plasma GSH

Group	Control		Patient		***p*** value
	Median	Range	Median	Range	
**ALT (U/L)** **N: up to 41**	25	21:30.8	51	35:67.5	< 0.001*
**AST (U/L)** **N: up to 40**	26	20:30	82	54:114	< 0.001*
**ALP (U/L)** **N: up to 279**	92.25	82:104.8	175	127:248	< 0.001*
**ALB (g/dl)** **N: 3.5:5.2**	4	3.9:4.2	3.27	2.8:3.5	< 0.001*
**INR** **N: up to 1**	1	1:1	1.24	1.1:1.3	< 0.001*
**UREA (mg/dl)** **N: 15-45**	31	28:36	28	22:34.5	0.007*
**AFP (ng/ml)** **N: up to 10.5**	1.85	1.2:2.7	117	16.3:1559.8	< 0.001*
**LDH (U/L)** **N: up to 250**	116.5	102.3:130	207	172:248	< 0.001*
**HB (gm/dl)** **N: Male (13:18)** **Female (12:16)**	12.3	11.1:13.4	13	12:14	0.001*
TLC*10^9/L **N: 4-11**	7.3	5.9:8.2	5.3	4.2:6.9	< 0.001*
PLT*10^9/L ***N: 150-400***	*226.5*	*194.3:280.8*	*125*	*81:195*	*<* 0.001*
**GSH (mM/mg)** **N: 684-2525**	2010	1619.8:2310.8	1916.5	(862-3011)	0.152

*INR* international normalization ratio, *ALT* alanine aminotransferase, *AST* aspartate aminotransferase, *LDH* lactate dehydrogenase, *AFP* alpha feto protein, *ALP* alkaline phosphatase, *GSH* reduced glutathione, *ALB* albumin, *HB* hemoglobin, *TLC* total leucocytic count, *PLT* platelets

*Significant *p* value ≤ 0.05

The association of the different genotypes of GSTP1 with some prognostic factors of HCC as age, gender, tumor size, number of focal lesions, stage, Child score, distant metastasis, portal vein thrombosis (PVT), splenomegaly, cirrhosis, ascites, lymph node metastasis, viral infection, and family history using chi-square test revealed non-significant results except for portal vein thrombosis which showed a significant association with GSTP1 genotype (*p* = 0.00783) (Table 5).

**Table 5:** Association of the different genotypes of glutathione S-transferase P1 with some prognostic factors of HCC using chi-square test

		GSTP1 homo hetero vs wild			***p*** value	
		Heterozygous variant	Homozygous variant	Wild		*X*^2^
		***N*** (%)	***N*** (%)	***N*** (%)		
**Age (years)**	**< 54**	20 (45.5)	8 (80)	23 (51.1)	0.142	3.899
	**≥ 55**	24 (54.5)	2 (20)	22 (48.9)		
**Gender**	**Female**	13 (29.5)	1 (10.0)	10 (10.0)	0.391	1.878
	**Male**	31 (70.5)	9 (90.0)	35 (77.8)		
**Tumor size**	**≤ 3 cm**	16 (36.4)	4 (40.0)	16 (35.6)	0.966	0.070
	**> 3 cm**	28 (63.6)	6 (60.0)	29 (64.4)		
**No. of focal lesions**	**1**	24 (54.5)	4 (40.0)	23 (51.1)	0.286	0.381
	**2**	3 (6.8)	3 (30.0)	8 (17.8)		
	**3**	4 (9.1)	1 (10.0)	1 (2.2)		
	**More than 3**	13 (29.5)	2 (20.0)	13 (28.9)		
**Stage**	**Early stage (I, II)**	12 (27.3)	3 (30.0)	11 (24.4.0)	0.918	0.172
	**Late stage (III, IV)**	32 (72.7)	7 (70.0)	34 (75.6)		
**Child score**	**A**	23 (53.3)	8 (80.0)	26 (57.8)		
	**B**	21 (47.7)	1 (10.0)	18 (40.0)		
	**C**	0 (0)	1 (10.0)	1 (2.2)		
**Distant metastasis**	**No**	42 (95.5)	8 (8.1)	36 (36.4)	0.077	5.118
	**Yes**	2 (4.5)	2 (2.0)	9 (9.1)		
**Liver cirrhosis**	**Negative**	1 (2.3)	0 (0.0)	1 (2.2)		
	**Positive**	43 (97.7)	10 (100.0)	44 (97.8)		
**Portal vein**	**Thrombosed**	6 (13.6)	6 (60.0)	8 (17.8)	0.004	9.700
	**Patent**	38 (86.4)	4 (40.0)	37 (82.2)		
**Splenomegaly**	**−ve**	15 (34.1)	5 (50.0)	17 (37.8)	0.642	2.037
	**+ve**	29 (65.9)	5 (50.0)	28 (62.2)		
**Ascites**	**Present**	16 (36.4)	4 (40.0)	20 (44.4)	0.739	0.292
	**Absent**	28 (63.6)	6 (60.0)	25 (55.6)		
**L.Ns**	**−ve**	39 (88.6)	8 (80.0)	26 (57.8)	0.004	4.905
	**+ve**	5 (11.4)	2 (20.0)	19 (42.2)		
**Family history of HCC**	**Negative**	37 (84.1)	7 (70.0)	43 (95.6)	0.048	5.286
	**Positive**	7 (15.9)	3 (30.0)	2 (4.4)		
**HBV infection**	**B−ve**	42 (95.5)	10 (100.0)	44 (97.8)	*	
	**B + ve**	2 (4.5)	0 (0.0)	1 (2.2)		
**HCV infection**	**C−ve**	6 (13.6)	0 (0.0)	10 (22.2)	0.187	3.355
	**C + ve**	38 (86.4)	10 (100.0)	35 (77.8**)**		

Significant *p* value ≤ 0.05

Number and percentage in parenthesis

*GSTP1* glutathione S-transferase P1, *GSTP1* glutathione S-transferase P1

*p value cannot be assessed due to small number

*Significant *p* value ≤ 0.05

The comparison of some laboratory markers as ALT, AST, bilirubin, ALP, albumin, AFP, LDH, PT, urea, creatinine, plasma level of GSH, hemoglobin, TLC, and platelet count according to GSTP1 genotype revealed non-significant results except for ALT. The median level of ALT was highest in the homozygous variant group followed by heterozygous variant, lastly, comes the wild type group with medians of (106.5, 51, and 43.5 U/L) respectively (*p* = 0.049) (Table 6).

**Table 6:** Comparison of some laboratory markers according to GSTP1 genotype

	Heterozygous variant	Homozygous variant	Wild	***p*** value
	Median (range)	Median (range)	Median (range)	
**ALT (U/L) (up to 45)**	51.0 (23-135) a	106.50 (54-181) b	43.50 (9-150) c	0.049*
**ALB (g/dl) (3.5:5.2)**	3.30 (1.8-4.87)	3.1 (2.7-4.2)	3.1 (2.2-4.3)	0.579
PLT*10^9/L (150-400)	101.5 (50-260)	102.5 (63-244)	120.000 (28-365)	0.422
**AST (U/L) (up to 41)**	79 (19.8-208)	69.5 (30-229)	73 (10-508)	0.698
**BIL (mg/dl) (up to 1.1)**	1.2 (0.7-3.82)	1.3 (0.2-5.8)	1.2 (0.5-4.9)	0.684
**ALP (U/L) (up to 279)**	165 (70-713)	207.0 (85-900)	150.0 (57-808)	0.543
**INR (up to 1)**	1.21 (1-2.5)	1.2 (1.00-1.31)	1.2 (1-1.8)	0.486
**Creatinine (mg/dl) (0.7-1.4)**	0.9 (0.6-1.5)	1 (0.80-1.2)	0.9 (0.5-1.84)	0.835
**UREA (mg/dl) (15-45)**	30 (17-60)	32 (19-50)	32 (17-71)	0.363
**AFP (ng/ml) (up to 10.5)**	103.4 (4.938295)	580.8 (2.3-155449)	165 (2.3-126817)	0.144
**LDH (U/L) (up to 250)**	190 (88-331)	209.5 (101-330)	206 (105-303)	0.012*
**HB (gm/dl)** **N: Male (13:18)** **Female (12:16)**	12.4 (8.2-15.7)	11.4 (9.3-14.8)	12.5 (8.9-15.9)	0.835
TLC*10^9/L (4.0:11.0)	4.7 (2.5-13.9)	4.6 (2.5-10.065)	4.8 (10.2-12.87)	0.683
**GSH (μM/mg) (684-2525)**	2000 (877-2911)	1507.5 (894-2241)	1851 (862-3011)	0.208
**PC (%)**	0.8 (0.4-79.5)	0.8 (0.7-1)	0.8 (0.5-67.5)	0.867

Median and range in parenthesis

Significant *p* value ≤ 0.05

*INR* international normalization ratio, *PC* prothrombin concentration, *ALT* alanine aminotransferase, *AFP* alpha feto protein, *GSH* reduced glutathione, *AST* aspartate aminotransferase, *ALP* alkaline phosphatase, *ALB* albumin, *LDH* lactate dehydrogenase

*Cannot be measured due to small no. of values

a, C non-significant (*p* value 0.886)

b, C significant (*p* value 0.049)

c, B non-significant (*p* value 0.162)

Comparison of ALT according to GSTP1 genotype shows a significant difference between the wild and mutant homozygous group (*p* value 0.049) while a non-significant difference was found between the wild and mutant group (*p* value 0.886) and between the mutant heterozygous and mutant homozygous groups (*p* value 0.162).

**Fig. 3:**
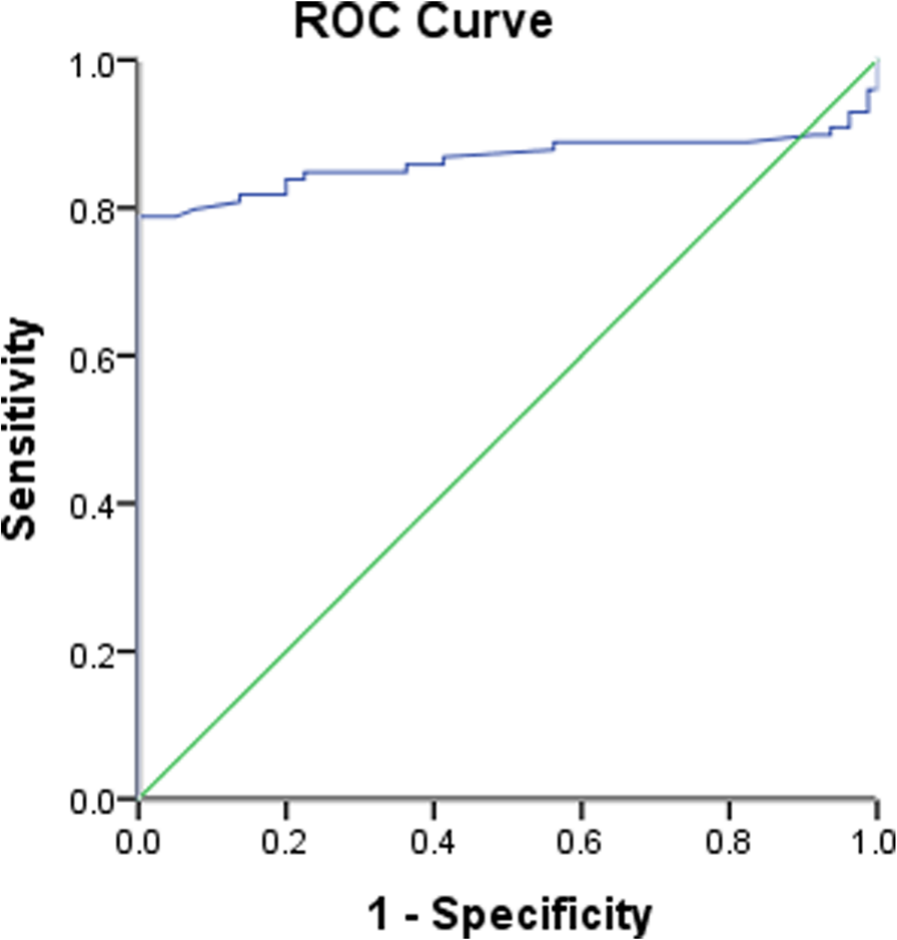
ROC curve analysis for AFP showing the best cutoff value to differentiate between the HCC and normal control groups. Area under the curve (AUC) was 0.864

**Fig. 4:**
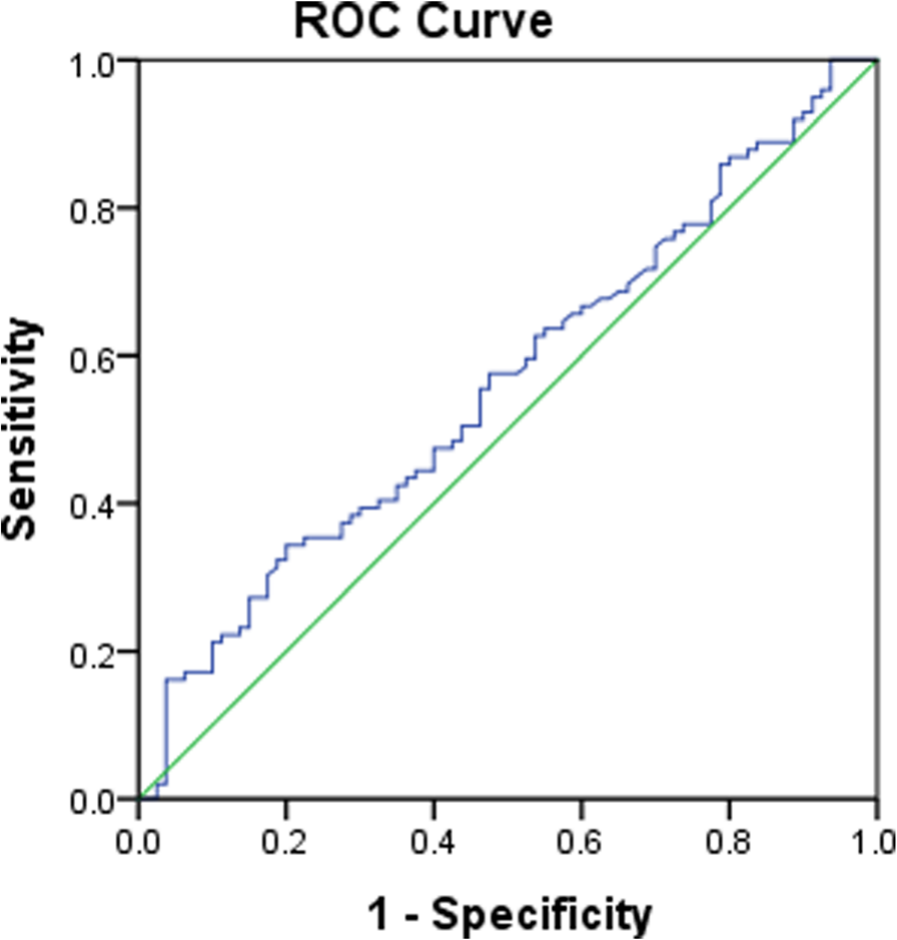
Roc curve analysis of plasma GSH showing the best cutoff value to differentiate between the HCC and normal control groups. Area under the curve (AUC) was 0.562

The best sensitivity, specificity, PPV, NPV, and overall diagnostic performance for plasma GSH at a cutoff level of 2003.5 μM/mg and for AFP at a cutoff of 21.5 ng/ml were 57.6%, 52.5%, 60%, 40% and 78.8%, 100%, 100%, 79.2%, respectively. The area under the curve for GSH is 0.562 and for AFP is 0.864 (Figs. 3 and 4).

On performing multivariate analysis for independent prognostic factors in relation to HCC risk, the mutant GSTP1 phenotype had 3.4-fold higher risk than the wild phenotype [*p* < 0.047, CI (1.015-11.386)], high serum ALT had 14.910-fold higher risk than low serum ALT [*p* < 0.001, CI (4.190-53.053)], male gender had 13.583-fold higher risk than female gender [(*p* < 0.001, CI (3.702-49.838)], and older age had 19.329-fold higher risk than younger age [*p* < 0.001, CI (5.633-66.755)].

GSTP1 gene for control fits the Hardy-Weinberg equilibrium with *p* value 0.124.

## Discussion

Because Egypt has the highest prevalence of hepatitis C virus HCV worldwide, the burden of HCC has been increasing with a doubled incidence rate in the past 10 years [11].

Continuous oxidative stress has been associated with hepatocarcinogenesis, suggesting that antioxidant treatment may provide some sort of protection against cancer [12].

Since the main action of the oxido-reductive enzymes (superoxide dismutase, catalase, and glutathione peroxidase) that constitute the most important scavenger systems for free radicals is to provide a steady supply of GSH, conjugate GSH with various environmental risk factors [13], as well as to control the action of specific transporters to remove GSH conjugate from the cell [6], a dramatic downregulation of such enzymes has been considered to be a characteristic pathological feature of HCC [14].

Hence, GSTP1 polymorphisms could decrease detoxification when individuals are exposed to HCC risk factors [15] so we hypothesized that GSTP1 polymorphism could be a probable risk factor for the development of HCC.

Also investigating the oxidative stress status of HCC patients by measuring the antioxidant glutathione (GSH) levels and assessing its relation with GSTP1 polymorphism were important objectives of this study.

In this study, 30% of the normal control and 54.4% of the HCC groups had mutant GSTP1 genotype, while 70% of the normal control and 45.5% of the HCC groups had the wild genotype (*p* = 0.0005).

Consistently, El-Shafie et al. [16] reported a significant difference between the HCC and the control groups regarding GSTP1 genotyping.

Also, Munaka et al. [17] detected the expression of mutant GSTP1 genotype in 33.3% and 23.1% and the wild genotype in 66.7% and 76.9% in the normal control and HCC groups respectively among the Japanese patients.

Mutant type of GSTP1, older age, male gender, and high serum alanine aminotransferase (ALT) were found to be significant independent prognostic factors for HCC risk in this study (*p* < 0.047, < 0.001, < 0.001 and < 0.001), respectively, which is contradictory to the results of Chen et al. [10], who found that individuals aged ≤ 57 years with AG or GG alleles of GSTP1 had a (2.18 and 5.64) fold risk of developing HCC compared to individuals with AA alleles (*p* = 0.02 and 0.04) respectively, but no association was found in the older group aged > 57 years. In the present study, patients and control subjects were selected below the age of 57 as we wanted to compare our patient’s population with the group studied by Chen el al [10].

Lu et al. [18] stated that the younger the age, the more likelihood for the exposure to HCC risk factors thus increasing the susceptibility to develop HCC. Since cirrhosis caused by hepatitis C virus infection is the most important predisposing factor for HCC in Egypt, the development of HCC can occur at a relatively older age as cirrhosis takes relatively long duration to turn into HCC.

On the contrary, Zhao et al. [19], found no association between GSTP1 polymorphism and HCC risk. Subgroup analyses by ethnicity showed no significant association between GSTP1 polymorphism and HCC risk among Asians [19] and Japanese people [17], and decreased risk among Europeans [14], while no association was detected among Chinese people who were exposed to high levels of aflatoxin B1 [20].

On studying the association between GSTP1 polymorphism and the clinical status of the HCC patients the only significant association detected with PVT (*p* = 0.00783).

Consistently, Chen et al. [10] found no association between the estimated clinico-pathological characteristics in HCC patients and gene polymorphisms of GSTP1.

ALT level was also found to be affected by GSTP1 phenotype (*p* = 0.049). The highest concentrations were detected among the mutant homozygous, followed by the mutant heterozygous, then the wild phenotype groups (*p* = 0.049).

Similarly, Mannaa et al. [21] found that increased serum levels of AST and ALT were highly correlated with the increased expression of GSTP1. On the other hand, Chen et al. [10] and Li et al. [22] found non-significant associations between genetic polymorphisms of GSTP1 and both ALT and AST.

ALT can produce pyruvate and l-glutamate by reversibly catalyzing the transfer of an amino group from l-alanine to α-ketoglutarate. l-glutamic acid, glycine, and l-cysteine are the amino acids needed for glutathione synthesis in the body. That is how ALT activity can affect the glutathione concentration inside the body [23].

During inflammation, GSH released from hepatocytes can detoxify reactive oxygen species (ROS) generated in the vascular space of the liver. However, diffusion of the ROS into hepatocytes can result from excessive ROS formation. This can lead to intracellular oxidant stress that can activate a series of transcription factors which can induce 500 genes’ expression; some of which could contribute to carcinogenesis [24] and can cause cell injury through mitochondrial dysfunction [25].

A higher level of GSH has got two contradictory actions: it is important for normal cellular functions, signal transduction, and protection against certain carcinogens, but at the same time can slow down any cancer therapy that works by increasing intracellular reactive oxygen species [25].

On the other hand, although an increase in the ROS in cancer cells is part of the carcinogenesis process, such excessive levels of the ROS can also be toxic to the cancer cells. Therefore, controlling the levels of intrinsic ROS by GSH modulation can be an effective way to selectively kill cancer cells without adversely affecting normal cells [26].

Considering the important role of GSTP-1 in the antioxidant defense mechanism, we further measured the levels of glutathione being an important antioxidant. Although the plasma levels of glutathione were lower in the HCC compared to the normal control group in this study, the comparison did not reach statistical significance (*p* = 0.156). We also could not find any significant relation between GSTP1 polymorphism and the plasma level of GSH (*p* value 0.569).

Consistently, Tsaiel al [27]. reported that the levels of GSH were significantly lower in patients with hepatitis B virus (HBV)-associated HCC than in the control group. Also, Czeczot el al [28]. found that GSH level was lower in malignant tissues compared to adjacent normal tissues.

Similarly, Li et al. [22] found that plasma GSH and GST levels were statistically lower in HCC patients than in chronic hepatitis C (CHB) patients. These results indicated that HCC patients are under more severe imbalance of oxidants and antioxidants than patients with benign liver conditions [24].

On the contrary, Huang et al. [4] demonstrated that GSH levels were doubled in the HCC compared to the normal group and Li et al. [22] found that GSTP1 protein expression level was significantly correlated with GSH concentration (*p* < 0.01).

We found that GSH concentration was highest in patients with Child score A, and patients with no ascites (*p* = 0.043) each. This might be due to the severe imbalance of oxidative stress and antioxidant defense among the patients with advanced disease status.

We could not find any significant relation between GSTP1 polymorphism and alpha-fetoprotein level in serum (*p* = 0.812). Similarly, Chen et al. [10] found no association between the gene polymorphism of GSTP1 and AFP.

Up to our knowledge, the clinical utility of the plasma GSH level in HCC was not previously studied. So, we tried to test for the validity of plasma GSH to be used as a diagnostic marker for HCC.

GSH and AFP showed sensitivity and specificity of (38%, 53%) and (79%, 94%) respectively. So, plasma levels of glutathione cannot be considered a probable candidate marker for the diagnosis of HCC; however, further studies with larger groups of population are recommended to validate such results.

Yusof et al. [29] estimated the sensitivity of AFP in HCC as 36.7% compared to 53.3% for GSTP1, thus making GSTP1 a more sensitive marker for detection of HCC.

## Conclusions

The prevalence of mutant GSTP1 was higher among the HCC patients compared to the normal control group, which might nominate GSTP1 to be a probable marker for screening of HCC among the highly susceptible individuals.

Based on our data, the mutant GSTP1 phenotype, older age, male gender, and high serum ALT are associated with an increased risk of HCC development. Thus, GSTP1 gene polymorphism is recommended to be incorporated in the risk assessment model which would allow early diagnosis, better management, and use of probable targeted therapy in selected patients.

Further studies with larger numbers of patients are recommended to validate these results.

## Data Availability

Available

## Abbreviations

HCC: Hepatocellular carcinoma
GSH: Reduced glutathione
GSTP1: Glutathione S-transferase P1
PCR-RFLP: Polymerase chain reaction-restriction fragment length polymorphism
PVT: Portal vein thrombosis
ALT: Alanine aminotransferase
GSSG: Oxidized glutathione
GSR: Glutathione reductase
GSTs: Glutathione *S*-transferases
ROC: Receiver operating characteristics
ROS: Reactive oxygen species
DNA: Deoxyribonucleic acid
PCR: Polymerase chain reaction
AST: Aspartate aminotransferase
ALP: Alkaline phosphatase
PPV: Positive predictive value
NPV: Negative predictive value
